# TBC1D14 sets the TRAPP for ATG9

**DOI:** 10.1080/15548627.2016.1177696

**Published:** 2016-05-12

**Authors:** Christopher A. Lamb, Sharon A. Tooze

**Affiliations:** The Francis Crick Institute, Molecular Cell Biology of Autophagy, Lincoln's Inn Fields Laboratories, London, UK

**Keywords:** ATG9, autophagy, endosome, golgi, RAB protein, TBC1D14, TRAPP

## Abstract

Amino acid withdrawal induces the formation of autophagosomes, which results in dozens of these large double-membrane vesicles appearing in the starved cell within 10–15 min, and the initiation of autophagy. This vesicle-mediated response clearly requires an adequate supply of membrane and a tight molecular regulation creating a substantial challenge for the cell in terms of vesicle trafficking pathways. Several membrane sources, which contribute to autophagosome initiation and formation, have been identified including the ER, Golgi, plasma membrane, mitochondria and recycling endosomes. How contributions from these organelles are regulated is an intensive area of study. Members of several families of membrane traffic regulators, including small GTPases, such as RAB proteins, and their regulators, SNARE proteins and BAR domain-containing proteins, have recently been shown to support autophagosome formation.

To uncover RAB protein-dependent roles during macroautophagy/autophagy, we carried out an overexpression screen of TBC (Tre-Bub-Cdc16 domain) proteins, which are predicted to harbor RAB GTPase activating protein (GAP) activity. In particular, TBC1D14 overexpression negatively regulates autophagy in HEK293A cells. Although we found TBC1D14 overexpression affects autophagosome formation and also has profound effects on recycling endosome structure and function, we could not identify any GAP activity associated with the protein. In fact, TBC1D14 is likely to act as a RAB11 effector, preferentially binding to a GTP-activated form of RAB11.

In our recent study, we used a mass spectrometry approach to identify membrane-trafficking regulators binding to TBC1D14 and identified several members of the mammalian TRAPP (TRAfficking Protein Particle) complex. TRAPP complexes are multi-subunit tethering complexes conserved in eukaryotes that act as GEFs (GTP exchange factors) for RAB1, the ortholog of yeast Ypt1. Yeast TRAPP complexes are well characterized, with 3 complexes known to play different roles in membrane traffic. TRAPPI mediates ER-Golgi transport, and TRAPPII regulates endosome-Golgi transport. TRAPPIII, via its Trs85 subunit, has been linked to autophagy in several recent studies. TRAPPIII is thought to be required for recruitment of Ypt1-positive, Atg9-containing vesicles to the phagophore assembly site, or preautophagosomal structure (PAS), resulting in the formation of the phagophore. More recently, TRAPPIII has been shown to be required for retrieval of Atg9 from the endosomes to the Golgi, resulting in a mobile “store” of Atg9 vesicles that can be recruited for PAS and phagophore formation upon starvation (Fig. 1A).

**Figure 1.**
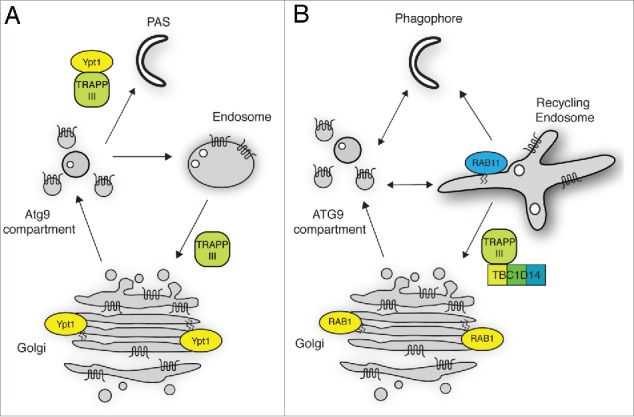
ATG9 traffic in yeast and mammalian cells. (A) In yeast, Atg9 trafficks between endosomal compartments, the Golgi and the Atg9 peripheral sites (Atg9 compartment) all of which may act as a readily mobilizable reservoir. Retrieval from early endosomes to the Golgi is regulated by the Trs85-containing TRAPPIII complex. Upon starvation, several Atg9 vesicles undergo homotypic fusion, forming the phagophore. This event depends on TRAPPIII and Ypt1. Atg9 persists in the PAS and autophagosome until fusion with the vacuole (not shown). (B) In mammalian cells, ATG9 cycles between endosomes (including recycling endosomes), the Golgi and the ATG9 compartment. ATG9 retrieval to the Golgi from RAB11-positive recycling endosomes to RAB1-positive Golgi compartments is controlled by a complex including TBC1D14 and mammalian TRAPPIII. This process generates a cycling pool of ATG9 similar to that seen in yeast. Upon starvation, ATG9 vesicles traffic to and from the phagophore to support autophagosome formation, without integrating into the phagophore membrane.

In mammals, the role of TRAPP in autophagy is less clear. Thus, we sought to dissect the function of the complex by studying the interaction between TBC1D14 and TRAPP to better understand how TRAPP works during mammalian autophagosome formation. We confirmed the interaction between TRAPP and TBC1D14 using co-immunoprecipitation, and also showed that TRAPP subunits colocalize on recycling endosome tubules induced by TBC1D14 overexpression. Intriguingly, RAB1 is also found on these tubules, suggesting they may form as a result of inappropriate RAB1 activation by mislocalized TRAPP.

We identified an N-terminal region in TBC1D14 required for TRAPP binding. Overexpression of this TRAPP binding region (TBR) alone does not affect recycling endosome function but does result in Golgi fragmentation and inhibition of constitutive secretion. This finding supports the idea that the TBR may sequester TRAPP subunits and prevent their normal function, as TRAPP depletion affects Golgi structure.

To identify which subunit of TRAPP may be mediating the interaction between this complex and TBC1D14, we employed a proximity-dependent biotinylation technique known as BioID, followed by mass spectrometry. This revealed that TRAPPC8 is the most proximal TRAPP subunit to TBC1D14, and that TRAPPC8 is required for the rest of the TRAPP complex to interact with TBC1D14. Intriguingly, TRAPPC8 is the mammalian ortholog of Trs85. Our additional data indicate that TRAPPC8 forms part of a mammalian TRAPPIII-like complex, required for RAB1 activity. Depletion of TRAPPC8 results in similar membrane-trafficking phenotypes to TBR expression, with fragmented Golgi and decreased constitutive secretion. Indeed, both TBR expression and TRAPPC8 depletion inhibit autophagosome formation at an early stage, as the number of starvation-induced GFP-ZFYVE1/DFCP1, WIPI2 and GFP-LC3 spots are all reduced.

Given the role of Trs85 in autophagy and Atg9 traffic in yeast, we investigated how TBR expression or TRAPPC8 depletion affects ATG9 traffic. ATG9 is found in a juxtanuclear compartment colocalizing with the Golgi, but upon starvation this pool redistributes to endosomal compartments, and this redistribution is inhibited in HEK293A cells lacking ULK1. Interestingly, loss of TRAPPC8 or overexpression of TBR lead to disruption of the Golgi pool of ATG9 in fed cells. This disruption is still evident in TBR-expressing cells lacking ULK1, indicating that the TRAPPIII-TBC1D14 axis controls a ULK1-independent constitutive ATG9 trafficking step.

Our study provides the first characterization of the role of a mammalian TRAPPIII-like complex and its GEF activity in starvation-mediated autophagy. Importantly, our study identifies parallels but also differences between ATG9 trafficking in yeast and mammalian cells. Our previous work showed that ATG9 vesicles do not nucleate or integrate with phagophore membranes, unlike yeast where this process is regulated by TRAPPIII and Ypt1 (Fig. 1A). Our new data support a model where mammalian TRAPPIII regulates the cycling of ATG9 between endosomes and the early Golgi, providing a ready supply of ATG9 from either compartment, or the ATG9 compartment, to support autophagosome formation both on induction of, and during, starvation (Fig. 1B). This model has some similarity with the yeast mobile “reservoir” model, with Atg9 traffic through endosomal compartments and retrieval to the Golgi stack being crucial for autophagy.

Recent proteomics studies of yeast Atg9 vesicles have revealed novel regulators of Atg9 traffic, including the TRAPPs. Given our findings on ATG9 and TRAPP, it will now be of great interest to expand similar studies with mammalian ATG9 vesicles to reveal further regulatory factors, including proteins and lipids. These studies may help to explain why mammalian ATG9 does not integrate into the forming phagophore, unlike what is proposed for the yeast counterpart, and further our understanding of how ATG9 traffic is regulated in mammalian cells.

